# Adapting to sea level rise: participatory, solution-oriented policy tools in vulnerable Mediterranean areas

**DOI:** 10.1007/s10669-023-09910-5

**Published:** 2023-06-01

**Authors:** Xenia I. Loizidou, Demetra L. Orthodoxou, Michael I. Loizides, Demetra Petsa, Marco Anzidei

**Affiliations:** 1 Isotech Ltd Environmental Research and Consultancy, Nicosia, Cyprus; 2 Istituto Nazionale di Geofisica e Vulcanologia, Rome, Italy

**Keywords:** Decision-making, Climate change and adaptation, Stakeholder engagement, Sea Level Rise, Mediterranean

## Abstract

The coasts of the Mediterranean basin are exposed to the ongoing effects of climate change and anthropogenic pressure. Low elevated coastal plains, river deltas, lagoons and reclamation areas are experiencing beach retreat, coastal erosion and marine flooding. This makes them particularly vulnerable to sea level rise (SLR), which is expected to increase up to 1 m by 2100 AD, according to the projections of the Intergovernmental Panel on Climate Change. In this study, selected stakeholders from four Mediterranean coastal areas that are highly vulnerable to the impacts of SLR have been engaged through a structured participatory process for the development of solution-oriented, case-specific and site-specific Policy Tools to address SLR. The developed Policy Tools for the Venice Lagoon, the Metaponto reclamation area and the Basento river mouth, in Italy, the Ebro River Delta in Spain, and the coastal plain of Chalastra, near the Axios River Delta, in Greece, contain relevant, effective and implementable actions stemming from stakeholder interaction and consensus building. The interconnected stakeholder engagement steps employed in this study identified relevant issues that should be considered when defining SLR adaptation policies to bridge knowledge and perception gaps, facilitate knowledge exchange and foster social learning through structured science communication on SLR. This participatory stakeholder process can lay the foundations for more extensive participation in public processes through which the resulting Policy Tools can materialise into collectively accepted, concrete actions to help vulnerable areas adapt to the expected SLR and consequent coastal hazards by the end of this century.

**Supplementary Information:**

The online version contains supplementary material available at 10.1007/s10669-023-09910-5.

## Introduction

Sea level data from global tidal gauge networks, space observations using radar altimeters and ground observations agree that sea level has been accelerating since the mid-nineteenth century and is currently rising at 3.7 mm/year, corresponding to a rate more than twice that of the twentieth century (Nerem et al. 2018; Fox-Kemper et al. 2021; IPCC AR5 Report 2014; Palmer et al. 2021). While there are natural causes for local deviations from global SLR, such as vertical land movements along the coasts due to tectonic and volcanic activity and glacial isostatic adjustment (Lambeck et al. 2010), global warming caused by the release of greenhouse gases from human activities is the main cause of this rise, as it triggers the melting of ice and the thermal expansion of the oceans (Oppenheimer et al. 2019; WCRP Global Sea Level Budget Group 2018).

SLR projections by 2100 AD (Fox-Kemper et al. 2021; www.ipcc.ch) predict a likely rise relative to the period 1995–2014 in the range of 0.28–0.55 m for a very low emissions scenario (66% confidence SSP1-1.9), 0.44–0.76 m for an intermediate emissions scenario (SSP2-4.5) and 0.63–1.02 m for a very high emissions scenario (SSP5-8.5), thus representing a factor of hazard for many coastal populations. The eventual fast melting of the Antarctic ice sheet could trigger a SLR up to 2.3 m by 2100 and up to 5.4 m by 2150 (Bakker et al*.*, 2017; Kopp et al., 2017; DeConto et al. 2021; https://www.eea.europa.eu/ims/global-and-european-sea-level-rise).

In the enclosed basin of the Mediterranean Sea, SLR by 2100 AD is estimated at about 60 cm (Aral and Chang 2017) but with large variability along its coasts due to the contribution of land subsidence and uplift caused by tectonics, volcanic activity and global isostatic adjustment (Anzidei et al. 2014). Land subsidence is a well-known issue along the coasts of the Mediterranean Sea, causing the submergence of ancient coastal settlements (Benjamin et al. 2017), historical cities like Venice, in Italy (Carminati et al. 2003) and small islands (Anzidei et al. 2017). The main drivers are long lasting tectonics (Douwe et al. 2020), volcanic activity (Marino et al. 2022) and exploitation of fluids from the ground (Carbognin et al. 2004; Vörösmarty et al. 2009). Major river deltas and reclamation areas are among the most vulnerable coastal zones to SLR (Galeotti 2020) because they are naturally subsiding due to their geological features (Syvitski et al. 2009), favouring soil compaction and therefore causing them to sink. These zones are often highly populated with productive ecosystems, closely connected with human activities. High rates of land subsidence can dramatically accelerate the rise of sea level in these areas, increasing their exposure to flooding and inundation, which may also occur in combination with high water levels during high tides and extreme meteorological events, threatening the living conditions of local populations. Because river deltas, lagoons and reclamation areas are transition zones between inland and coastal environments, they are highly sensitive to physical and biological changes that may further influence the integrity of water tables and the coastal zone dynamics at all levels. The anthropogenic factor makes these areas highly susceptible to infrastructure and ecosystem degradation due to industrialisation and pollution.

For the above reasons, SLR is having an unprecedented environmental and socio-economic impact on coastal populations (Tay et al. 2022). Salinisation due to the infiltration of salt water in the water tables because of SLR, land subsidence and coastal retreat are responsible for 85% of the damage costs along the Mediterranean coasts (Bouda et al. 2017), especially in the western Mediterranean sector of this basin (ten Veldhuis et al. 2011). The projected economic loss related to the loss of coastlines and their natural and cultural heritage in Southern Europe is estimated at EUR 18 billion for the period 1908–2080 (Joint Research Centre 2017). Since many countries in the Mediterranean Sea are heavily dependent on tourism and other coastal activities (e.g. agriculture, farming and maritime industry), the social and economic impacts of SLR are expected to be significant. For example, in Catalonia SLR is projected to lead to a decline of 20% in the area’s tourism-related GDP (Garola et al. 2022), whereas rice production in the Ebro River Delta is expected to decrease significantly, reducing farmers’ profits by up to 300 Euro per hectare (Genua-Olmedo et al. 2016).

Still, the associated risks are not well understood, at least not to the extent necessary for raising adequate awareness and implementing appropriate mitigation and adaptation policies. A more consolidated approach among scientists, decision-makers and stakeholders involved in managing the various aspects and effects of climate change and SLR is needed to fill the gap between science and policy (Mastrandrea et al. 2010; Santos et al. 2014; Kirchhoff et al. 2015). Furthermore, there is a lack of local stakeholder involvement in decision-making regarding SLR, as decision-makers and land planners often fail to recognise the importance of their knowledge and understanding of the local environmental and socio-economic characteristics. This results in local stakeholders feeling disempowered, or even removed, from the causes, impacts and solutions to SLR and climate change.

To develop sound and publicly accepted solution-oriented policies, it is important to engage the various stakeholders in structured decision-making processes, to understand their perceptions, listen to and record their area’s needs and encourage them to identify site-specific solutions (Burger et al. 2016; Stringer et al. 2007; Loizidou et al. 2016; 2017; 2021). Effective stakeholder participation can align stakeholder needs and values with necessary performance of coastal systems and create shared values and benefits (Ciampa et al. 2021). Participatory decision-making leads to social learning and can generate new knowledge, build capacity and foster trust and collaboration between stakeholders (Muro and Jeffrey 2008). This is particularly important for issues such as SLR and climate adaptation, where the direct and indirect impacts on people and the environment can be severe, where awareness is still relatively low, and where cross-sectoral competition for resources and funding could be significant and result in different preferences for action (Pasquier et al. 2020). The involvement of stakeholders in decision-making regarding adaptation to climate change and SLR empowers societal actors to work together towards making their areas more resilient, to overcome severe effects (Yusuf et al. 2018).

However, it must be considered that impacts from SLR and extreme weather conditions are not uniform in space and time. They are site-specific and therefore, require site- and case-specific approaches. Here, we show the results obtained by the SAVEMEDCOASTS-2 Project (www.savemedcoasts2.eu) through the implementation of an innovative method for stakeholder engagement in participatory decision-making. Through semi-structured interviews and dedicated workshops, local stakeholders were engaged in a process that allowed the identification of site-specific solutions to adapt to SLR and extreme weather conditions and mitigate their impacts.

## The study areas

**Fig. 1:**
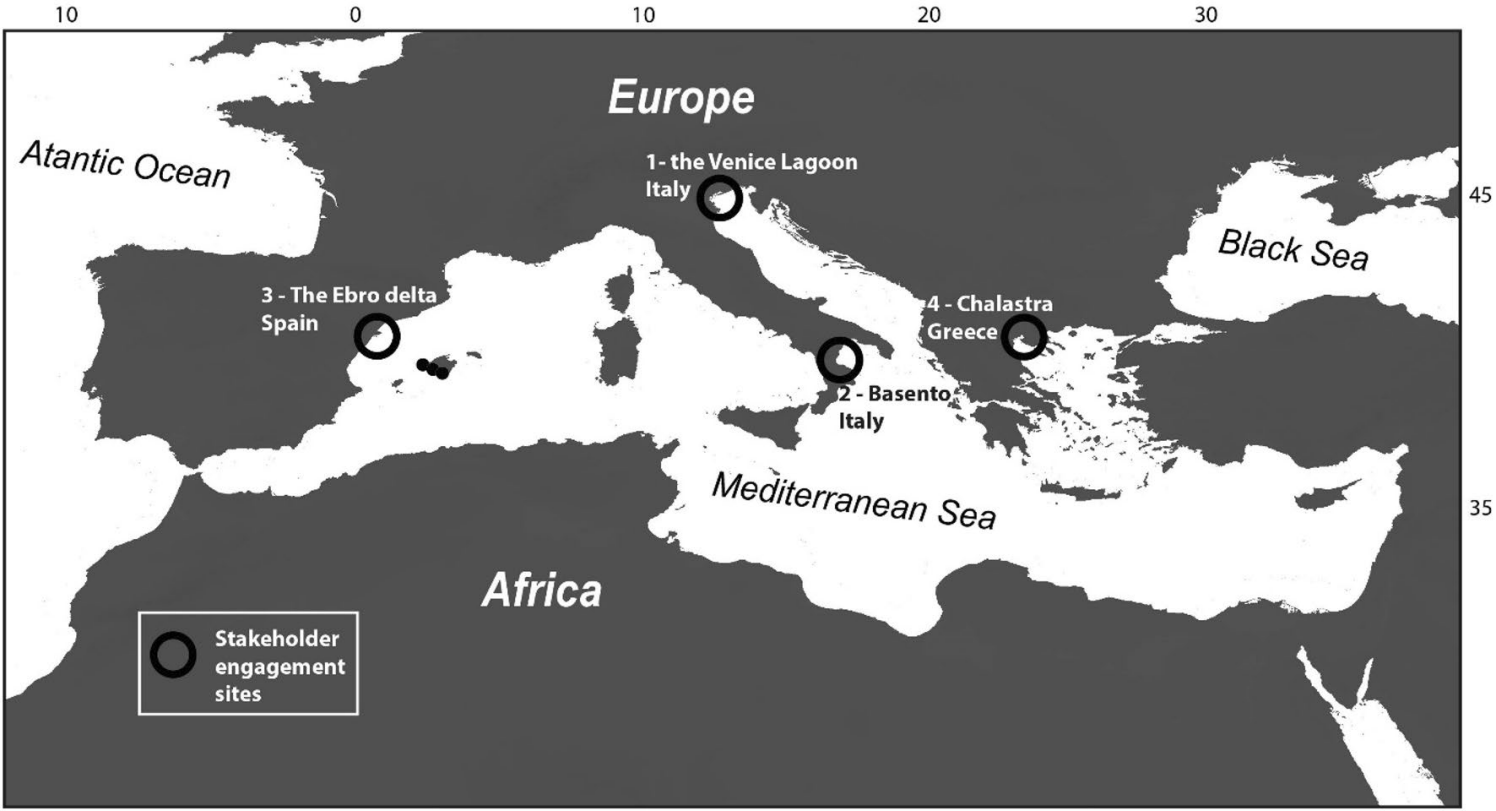
Map of the location of the four sites of stakeholder engagement

The study was implemented between 2019 and 2022 in the framework of the SAVEMEDCOASTS-2 project (www.savemedcoasts.eu), after a first experimentation carried out in the SAVEMEDCOASTS Project (www.savemedcoasts.eu). Stakeholder engagement was implemented in four areas of the Mediterranean basin that are highly vulnerable to SLR: the Venice Lagoon in Italy, the Metaponto reclamation area and the Basento river mouth, in Italy, the Ebro River Delta in Spain, and the plain of Chalastra, near the Axios River Delta, in Greece (Fig. 1).

### The Venice lagoon

The Venice lagoon is located in the north Adriatic Sea (Italy) and is a UNESCO world heritage site (https://whc.unesco.org/). It covers an area of about 550 km^2^, 155 km^2^ of which is land. About 62% of the area is of high ecological value. Around 260,000 people live in the lagoon; over 60,000 in the historical city of Venice, 27,000 on the islands and littorals, and the rest on the mainland (data from Comune di Venezia, 2021). Approximately 30 million tourists visit Venice every year putting intense pressure on infrastructures and services (City of Venice 2021). This coastal city is affected by the combined effects of SLR and natural and anthropogenic land subsidence as well as by frequent events of “acqua alta” (extreme high water levels due to the combined effect of tides and low atmospheric pressure). The frequency of extreme high water events in the last few decades has increased due to progressive SLR caused by global warming (Città di Venezia 2022b). When the high water reaches the elevation of 110 cm, about 12% of the historic city is flooded, while a height of 140 cm causes the flooding of 59% of the inhabited areas. Between 2019 and 2021, about 13 annual events with tide amplitudes ≥ 110 cm were recorded (Città di Venezia 2022a). The phenomenon has been amplified in time from natural and anthropic land subsidence caused by the extraction of groundwater and fluids between 1930 and 1970. Since the early 1900s, the mean sea level in Venice increased about 35 cm, higher than the 18 cm mean SLR of the Mediterranean in the same period (Zanchettin et al. 2009; 2021; Anzidei et al. 2014; Wöppelmann and Marcos 2012), thus putting the lagoon at increasing risk of flooding by 2100 due to SLR (Vecchio et al. 2019; Lionello et al. 2021).

### Metaponto and Basento

The second case study area is the coast of Basento and Metaponto, located along the Ionian coast of the Basilicata Region, in southern Italy. Approximately 1000 people live at Metaponto Lido (the part of the city nearest to the coast) and about 12,000 people in the town of Bernalda (data from ISTAT 2021). The area is an important cultural and historical site, as Metapontum was part of Magna Grecia and it is a popular touristic destination. The coast suffers severe erosion that is causing a beach retreat of approximately 2 m/year (Corbau et al. 2022; Greco and Martino 2014a). More compounding factors, including the extraction of material from the riverbed of the Basento river, the construction of upstream dams, human activity along the coast and coastal protection works are accelerating coastal erosion and beach retreat (Borona et al. 2002; Simeoni et al. 2003; Guariglia et al. 2006). In 2011, 9 million Euro were invested in replenishing the coast (Greco and Martino 2014a). A set of large concrete boulders were placed along the east coast of Metaponto to reduce coastal erosion and protect existing infrastructure. However, this resulted in the accelerated erosion of a coastal strip of 200 m long and 100 m wide, while the breakwaters built near the coast collapsed and were submerged in a short time (Greco and Martino 2014b).

### The Ebro delta

The Ebro Delta is located along the coast of Catalunya (Spain). It comprises an area of approximately 368 km^2^, which is inhabited by about 62,000 people and hosts nearly 442,000 tourists per year. Around 80% of the area is agricultural/farming land, while an area of 56 km^2^ is maintained as wetlands (Ibáñez and Caiola 2016). The main economic sectors in the Ebro Delta are rice cultivation and mollusc aquaculture. The 6-km-long isthmus of Barra del Trabucador extends to the southwest of the Delta, while the flat peninsula of El Fangar, with an extension of 410 ha, extends into the sea, north of the mouth of the Ebro River, offering protection to the mollusc cultivations. Both these areas, which lie less than 2 m above sea level, are highly vulnerable to storms and tides. In addition, they are undergoing coastal retreat and erosion due to the combined effects of land subsidence, SLR and fluvial sediment deficiency after the construction of two dams in the lower Ebro River that reduced sediment transport and deposition (Genua-Olmedo et al. 2016). In 2020, storm “Gloria” flooded and fragmented the Barra del Trabucador (Rodríguez-Santalla and Navarro, 2021). Local SLR in the Ebro Delta is estimated at about 4 mm/year, while the average subsidence is about 1–2.3 mm/year (Rodriguez-Lloveras et al. 2020) and the coastline regression has been estimated at 6–7 m during the last decades.

### Chalastra plain

The village of Chalastra, together with the nearby Kalochorion, are located in the plain of the Axios Delta, in the Thermaikos Gulf, within the jurisdiction of the Municipality of Delta, in Greece’s Prefecture of Central Macedonia. This area is part of the European Ecological Network of NATURA 2000 Areas, a network of nature protection zones in the territory of the European Union. The area of concern for this research, the Chalastra Plain, extends for 309 km^2^ and has a population of around 46,000 people. The main economic activities in the area are rice cultivation and mussel farming, covering 59% of the area, whereas 33.5% of the area is of high ecological value. The area is highly vulnerable to floods because the Axios river flows in a poorly sloping area, thus prone to frequent river overflows in case of heavy rains. In addition, significant land subsidence up to 1.2 cm/year is exacerbating the effects of SLR and preventing the outflow of the rivers in the Mediterranean Sea (Elias et al. 2020).

## Method

There are several participatory techniques identified in literature, each resulting in a different level of stakeholder engagement and involvement (Luyet et al. 2012). For this study, the DeCyDe-4 method and tools were implemented to engage stakeholders in the decision-making process concerning coastal hazards resulting from SLR and extreme events such as storm surges. DeCyDe-4 was developed by ISOTECH Ltd’s experts in participatory decision-making and stakeholder involvement facilitation. It is an adaptable, site- and case-specific decision-support method that leads to informed, science-based and justifiable decisions on issues relating to sustainability and resilience. The method has been extensively described by Loizidou et al. (2016; 2017; 2021) and Schumacher et al. (2018). The initials ‘DeCyDe’ are a play on the word ‘decide’, where the ‘ci’ has been replaced by ‘Cy’, which stands for Cyprus, the country where ISOTECH is based. The suffix ‘-4’ stands for the word ‘for’ and is included to denote the fact that the main “DeCyDe” method is adapted to meet the specificities of each decision-making problem at hand, forming dedicated “DeCyDe-4” tools. In this specific study, DeCyDe-4 was adapted to develop the DeCyDe-4-SLR version and associated tools, by incorporating stakeholder interviews in the engagement method, and including data and information specifically relating to SLR in the developed tools.

The specific aim of DeCyDe-4-SLR is to transfer scientific knowledge to stakeholders and facilitate them to make informed decisions about preventive and responsive measures that can be taken to mitigate and protect their areas from SLR and extreme events such as flooding and storm surges (henceforth referred to as SLR). It should be noted that in this study the term “mitigate” is used in the sense of reducing the impacts of SLR.

**Fig. 2:**
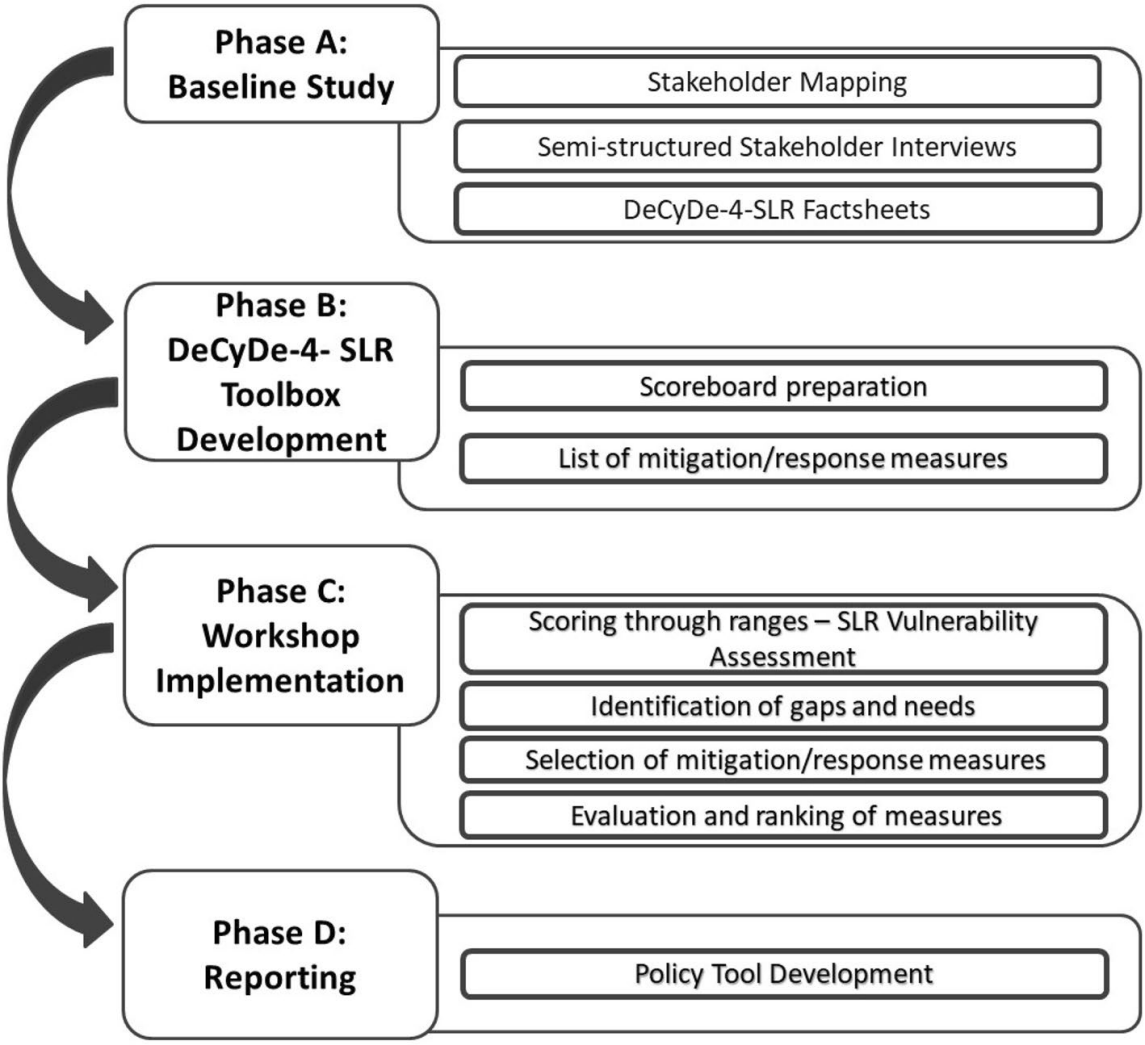
Graphical representation of the DeCyDe-4-SLR process

DeCyDe-4-SLR was implemented in four phases (Fig. 2). Phase A consists of the Baseline Study where stakeholders in each area are mapped, basic perceptions are recorded through semi-structured interviews and relevant information on the area are recorded through factsheets. Phase B concerns the development of a set of tools that are used for the successful implementation of DeCyDe-4-SLR. Phase C concerns the implementation of the participatory stakeholder workshops. Finally, all the outputs are brought together in Phase D for the development of the Policy Tools.

### Phase A: baseline study

Phase A of the DeCyDe-4-SLR method consists of establishing the baseline for each case study area. Specifically, the stakeholders in each area are mapped, basic perceptions are recorded through semi-structured interviews and relevant information on the area are recorded through factsheets.

#### Stakeholder mapping

Phase A of the DeCyDe-4-SLR method identified the relevant stakeholders among political, industrial, touristic, etc., for each of the investigated sites. An approach similar to the Prospex-CQI method (Gramberger et al. 2015) was used, which consists of (1) defining the Criteria (C) meaning the main categories of stakeholders that affect the topic of research (in this case SLR) or are affected by it, (2) setting specific minimum Quotas (Q) for these categories and (3) identifying Individuals (I) that fall within the categories and fulfil the required quotas.

Within this study, ten main categories of stakeholders were identified, further split into sub-categories (Table S1 in Supplementary Material), designed to include the following:

- Sectors that are affected by SLR in the Mediterranean coastal zones,
- Sectors that can (directly or indirectly) impact policymaking and decision-making with regards to SLR in their area,
- Sectors that have a role to play in raising awareness about SLR.

Rather than assigning quotas, it was considered more pertinent to assign priorities of stakeholder categories. Government representatives (at the local or national level), environmental organisations, civil protection organisations and representatives from the main economic sectors active in each area were considered priority sectors. Emphasis was placed on identifying non-conventional and grassroot level stakeholders.

The following criteria were used for the identification of individuals within the stakeholder categories and sub-categories:

- Individuals with sufficient knowledge and interest in the topic of SLR.
- Individuals with a decision-making role in their organisation.
- Individuals that are sufficiently aware of the impacts and effects of SLR in the sector they represent and the actions that are being taken within the sector to address these impacts.

#### Semi-structured stakeholder interviews

As part of Phase A of the method, the perceptions of a selection of key stakeholders from each of the study areas were explored through semi-structured interviews. A set of predefined questions and open discussion, focussed on exchanging knowledge and allowing information flow, were used. Semi-structured interviews were selected as they can produce comparable data (Reed et al. 2010), while at the same time they are flexible enough to allow “the researcher to probe for more detailed responses” (Gray 2004). Information about the purpose and process of the interview was provided to the identified stakeholders and an appointment was booked. The interviews were led by an experienced facilitator, member of the study team, who supported the stakeholders to share their perceptions related to SLR. The interviews began with an ice-breaking question to introduce the interviewees and to focus on SLR. Specifically, interviewees were asked to state the first word that came to their mind when they heard the term ‘SLR’. The interviews then included five sections, aiming to assess: (1) the major risks/impacts by SLR in the stakeholders’ country/area, (2) the actions already implemented or planned to address SLR in the stakeholders’ country/area, (3) the main needs in the stakeholders’ country/area with regards to SLR, (4) suggestions for further actions that could be implemented in the stakeholders’ country/area to address SRL and (5) the responsible bodies for mitigating/addressing SLR in the stakeholders’ country/area. Each interview took between 30 and 60 min. Interviews were not recorded to allow interviewees to express themselves more freely, however, detailed notes were taken by the research team. These were used to develop interview transcripts, which were then compared and compiled into one transcript per interview. The outputs, i.e. the perceptions on needs, solutions and responsibilities, that emerged from the interviews in each area, formed the basis for the implementation of the stakeholder workshops (Phase C of the method) as they provided a background on which to build and enhance the stakeholder engagement process.

#### DeCyDe-4-SLR factsheets

Phase A also included the development and completion of factsheets to record data and information to allow the assessment of the area’s vulnerability to SLR. The factsheets captured scientific and verifiable data and information across three Pillars: Natural Environment, Socio-Economic Parameters and Level of Preparedness. The Pillars relate to the four axes of sustainability: environment, society, economy and governance (introduced in the tool as ‘preparedness’). Each Pillar was defined by a set of Indicators, identified through literature and expert judgement (Table S2 in Supplementary Material).

### Phase B: DeCyDe-4-SLR toolbox development

In Phase B, scoreboards were developed to facilitate the assessment of vulnerability to SLR for each case study area. For each of the indicators included in the factsheets, “scoring ranges” were developed to allow the transformation of the indicator data recorded in the factsheets to numerical scores. Scoring ranges from literature were used (Table S3 in Supplementary Material) or, where these were unavailable, expert judgement was used to develop the scoring ranges. A score of either 1, 3, 5, 7 or 10 was assigned to each range (Table 1), where the lower the vulnerability to SLR the higher the assigned score. For indicators where only yes or no answers were possible, a score of 1 was assigned to yes and 10 to no. A score of 0 was assigned where no data were available to ensure that data gaps are reflected in the overall SLR vulnerability assessment. Once the scoring of indicators was completed, the average score for each Indicator Category and for each Pillar was calculated. The resulting scores were presented in a summary table format (Table 2), allowing the stakeholders to identify indicators with particularly low scores and focus on these for the identification of needs and solutions. The average Pillar score for each area was also translated into an overall SLR vulnerability level (Table 3).

**Table 1:** Example of part of the DeCyDe-4-SLR Self-assessment tool

1.1	Coastal area characteristics	Units	Scoring							Average Indicator Score
	1.1.1 Coastal elevation	m above sea level	Value ranges	< 0	0–0.5	0.5–1.0	1.0–1.5	1.5–2.0	No Data	3.00
			Score per value range	1	3	5	7	10	0	
			Assigned Score		**3**					
	1.1.2 Average coastal slope	%	Value ranges	< 0.6	0.6–0.9	0.9–1.3	1.3–1.9	> 1.9	No Data	
			Score per value range	1	3	5	7	10	0	
			Assigned Score	**1**						
	1.1.3 Land Subsidence	mm/year	Value ranges	> 12	8—12	4—8	0—4	< 0	No Data	
			Score per value range	1	3	5	7	10	0	
			Assigned Score		**3**					
	1.1.4 Coastal Erosion	m/year	Value ranges	> 0.30	0.20–0.30	0.10–0.20	0–0.10	0	No Data	
			Score per value range	1	3	5	7	10	0	
			Assigned Score			**5**

**Table 2:** Resulting table from the SLR vulnerability assessment, including the overall SLR vulnerability level of the area

Self-assessment and scoring for SLR vulnerability		
Pillars	Indicators	Indicator scores
1. Natural environment	1.1.1 Coastal elevation	0.00
	1.1.2 Average coastal slope	0.00
	1.1.3 Land subsidence	0.00
	1.1.4 Coastal erosion	0.00
	1.2.1 Frequency of extreme events	0.00
	1.3.1 Aquifers within the Area of interest	0.00
	1.3.2 River mouths within the area of interest	0.00
	**Average pillar score**	**0.00**
2. Socio-economic parameters	2.1.1 Resident population	0.00
	2.1.2 Tourism	0.00
	2.1.3 Tourism’s contribution to GDP	0.00
	2.2.1 Urban coverage	0.00
	2.2.2 Farming/agricultural coverage	0.00
	2.2.3 Areas of high ecological value	0.00
	2.3.1 Significant utility infrastructure	0.00
	2.3.2 Hazardous facilities	0.00
	2.3.3 Important emergency infrastructure	0.00
	2.3.4 Vulnerable infrastructure	0.00
	2.3.5 Economic impact of climate change-related damages	0.00
	2.4.1 UNESCO sites	0.00
	2.4.2 Distance of cultural heritage sites from coast	0.00
	**Average pillar score**	**0.00**
3. Level of preparedness	3.1.1 Policies/adaptive strategies	0.00
	3.1.2 Emergency plans	0.00
	3.1.3 Risk assessment	0.00
	3.1.4 Community awareness	0.00
	**Average pillar score**	**0.00**
Vulnerability score	0.00	
Vulnerability level	Extremely high

**Table 3:** SLR vulnerability score range and level

SLR vulnerability score	Level of vulnerability
9.51–10.00	Not vulnerable
8.51–9.50	Low
7.01–8.50	Moderate
5.01–7.00	Moderate-high
3.01–5.00	High
0.00–3.00	Extremely high

### Phase C: workshop implementation

Phase C concerned the implementation of the DeCyDe-4-SLR workshops with the participation of stakeholders, including decision-makers, identified through stakeholder mapping. Each workshop was designed to last a maximum of 3 h, during which the participants were guided by expert facilitators through a process that allowed them to:

- Undertake the SLR vulnerability self-assessment for their area, by converting scientific data into an overall SLR vulnerability score;
- Recognise the main gaps and needs in their area with regards to SLR;
- Identify and evaluate effective, applicable and socially acceptable solutions to address those gaps and needs.

The first step in this process, the completion of the self-assessment tool, allowed the participants to understand the importance of scientifically robust data for the evaluation of an area’s vulnerability to SLR and extreme weather conditions and to identify the indicators that have an effect on their increased vulnerability (i.e. the indicators where their area scores poorly). This part of the workshop was complemented by the projection of maps of potential land inundation scenarios for each area, based on the relative SLR projections of the IPCC (www.ipcc.ch) climate change scenarios (SROCC 2019 Report for RCPs 2.6 and 8.5) for 2030, 2050 and 2100. These maps, developed within the SAVEMEDCOASTS-2 project, further allowed the translation of scientific data into visual representations, raising knowledge and awareness among the stakeholders.

**Fig. 3:**
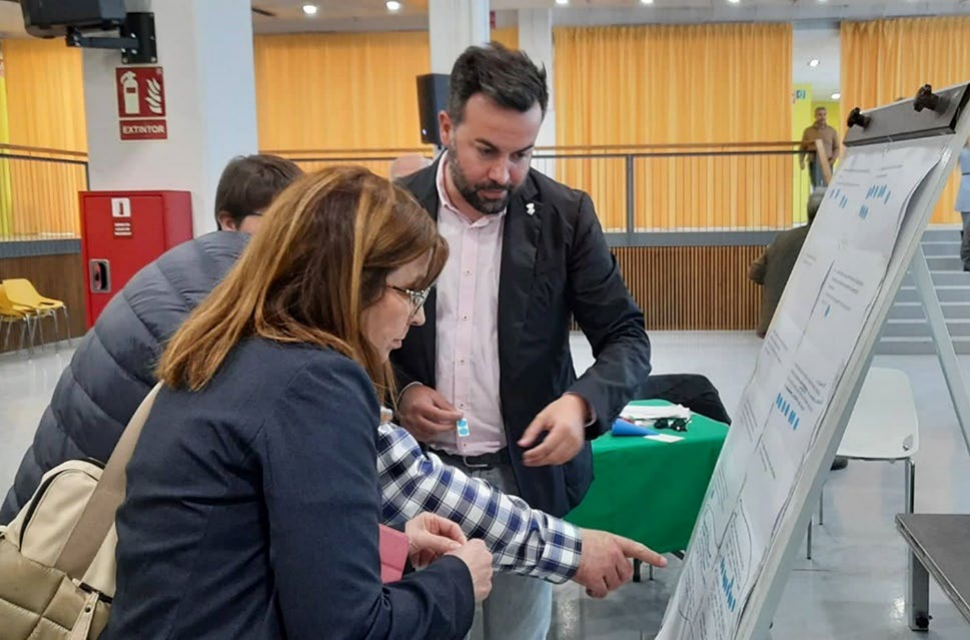
Stakeholders casting their votes on needs at the Ebro Delta Workshop in Deltebre, Spain

A collective intelligence exercise was then implemented to identify the specific needs of each case study area with regard to SLR. The needs emerging from the stakeholder interviews at each of the sites were presented to the participants. Working in groups and considering the results of the self-assessment and the potential land inundation maps, the participants were asked to consider whether there were additional needs to the ones identified during the interviews for their area with regard to SLR. These were added to the list of needs. Each participant was then given three coloured stickers and asked to vote the needs that s/he considered most important (Fig. 3). Participants were free to distribute their votes as they wished, by assigning one vote to three different needs or multiple votes to one need. At the end of this collective intelligence exercise, there was a ranked list of needs, based on stakeholder votes.

At each workshop, participants decided on the number of top-ranking needs to carry to the next step of the workshop for the identification of solutions. Participants then worked in groups to identify three possible solutions to address the top-ranking needs for their area. Participants worked on needs that were more relevant to their line of work/sectors. The identified solutions were evaluated through the DeCyDe-4-SLR multi criteria tool, by comparing couples as described in Loizidou et al. (2021) and Schumacher et al. (2018). This tool allows the ranking of the solutions by comparing each solution against all others in a pairwise manner in the format of a matrix. The comparison was done twice, once for each of the following two criteria:

- Implementation Potential: the ease with which the solution can be implemented in terms of coordination, time and resources required.
- Effectiveness: the extent to which the solution is effective in addressing SLR.

This part of the workshop resulted in a ranked list of solutions, at the top of which were those that participants considered to be most effective and applicable.

### Phase D: policy tool development

Policy tools or policy instruments are the means through which policy goals are met. In this specific case, the ultimate policy goal was adaptation and mitigation of the impacts of SLR and climate change. The Policy Tools developed within this study used the information stemming from the previous phases of the method to define an action plan responding to the most pressing needs identified at each site. The way forward addressing each important need has been defined in the Policy Tools incorporating the solutions identified by the stakeholders during the interviews and/or the workshops, and where necessary, complementing these through additional solutions and measures to ensure that a coherent action plan for each area is proposed.

## Results

The interviews took place in March–April 2021 by teleconference, due to COVID-19 travel-related restrictions, while workshops were implemented in person between October 2021 and May 2022. In Sect. 4.1, the engaged stakeholders through the interviews and the workshops are presented. Sections 4.2 to 4.5 show the gaps and needs, suggestions and policy tools for each study area resulting from the interviews and the workshops, as well as the developed policy tools for each area. The tables showing the results of the SLR vulnerability assessment, based on the self-assessment tool, are not included in this paper as they contain sensitive data on the potential vulnerability of certain areas. Instead, the overall vulnerability level is presented. Selected quotes from stakeholders are included in the analysis, italicised and in quotation marks.

### Engaged stakeholders

A total of 98 relevant stakeholders were involved in this participatory process for developing the solution-oriented policy tools for SLR at the four study areas: Venice and Basento in Italy, Chalastra in Greece and the Ebro Delta in Spain. 24 of them were engaged in the interviews while 74 participated in the four workshops. The names of the participating stakeholders are omitted for data protection reasons. Sectors represented by the involved stakeholders (interviews and workshops) are reported in Table S4 in the Supplementary Material.

### Venice (Italy): gaps, needs, suggestions and the policy tool

Six stakeholders from the city of Venice were interviewed online, whereas 18 stakeholders were involved in the participatory workshop, which took place on 26 October 2021. The vulnerability assessment resulted in a High SLR Vulnerability for the city of Venice.

The selected stakeholders identified 15 needs and gaps with regard to SLR and chose to work on the three top-ranking ones for the definition of solutions (Table S5 in Supplementary Material). The provision of more information, education and training about how to react in case of emergencies was the most important need according to the stakeholders. This is despite the fact that the Tidal Forecasts and Early Warning Centre of the City of Venice (https://www.comune.venezia.it/it/content/la-previsione) gives 24-, 48- and 72-h flooding predictions and warnings about these events through different communication channels (application, emails, SMS, telegram, sirens, https://www.comune.venezia.it/it/content/servizi-allertamento). The stakeholders considered the implementation of trainings, awareness-raising activities and emergency drills, particularly at schools, to be the best way of ensuring that people, both residents and tourists, know how to react in case of emergencies of flooding and extreme high water-level events. The stakeholders also identified the provision of more information and the implementation of awareness-raising campaigns to encourage people to implement best practices for mitigating climate change as a solution. The practice of training citizens on emergency plans was another identified solution, although it ranked third. This is because the stakeholders believed that the provision of equivalent training at schools would ensure that adults (i.e. the parents of these students) would also be indirectly trained.

At the time of undertaking the interviews and the workshop, the MoSE (Modulo Sperimentale Eletromeccanico, https://www.mosevenezia.eu/?lang=en), the Venice lagoon flood barrier system, was operational since October 2020 on a pilot basis for almost a year. It therefore featured prominently both during the interviews and during the workshop. The feelings about the MoSE among the stakeholders were conflicting. For example, while most stakeholders recognised its importance, they were also concerned about the “*excessive*” amount of money spent for its construction and maintenance (about 6.2 billion euros), and about the fact that this money was being diverted from other necessary works that could protect the city against the effects of SLR, such as for example the cleaning of canals that run across the city of Venice. There was also concern that while the MoSE “*has allowed the City to breathe a sigh of relief it might also lead to a general ‘relaxation’ about the issue of flooding, and Venetians could think that it is no longer a threat. However, the* MoSE *has not been designed to address SLR. It has been designed for flooding from high tides. Therefore, the* MoSE *cannot stay closed permanently*—*this would be catastrophic to the lagoon ecosystem and will affect the quality of life of the people. Even if MoSE were to stay closed, some lower parts of the city would flood anyway*”.

Therefore, managing and operating the MoSE in a faster and more flexible manner that would ensure that the barriers are active for as little time as possible, was identified as the second most important need by Venetian stakeholders. According to the stakeholders, this could be achieved through the better definition of the governance of MoSE, as this was considered to be the most effective solution. The improvement of the operating procedures for flexible, quick and effective management of the barriers, and the improvement of the communication between the various interested parties, including the local port authorities, whose operation is negatively impacted when the MoSE is activated, were also identified as suitable solutions.

Protecting vulnerable areas was the third most important need identified by stakeholders in Venice. The reinforcement of natural defences, such as the restoration of salt marshes and enhancing natural sand dune vegetation, was the top-ranking solution as it was considered particularly effective at protecting vulnerable coastal areas. Stakeholders considered that this solution could be supported by the implementation of nature-based solutions to protect vulnerable areas from SLR and storm surges. The Venetian stakeholders also proposed the implementation of studies to identify the best possible, environmentally sound solutions for addressing Venice’s water shortage problem.

**Fig. 4:**
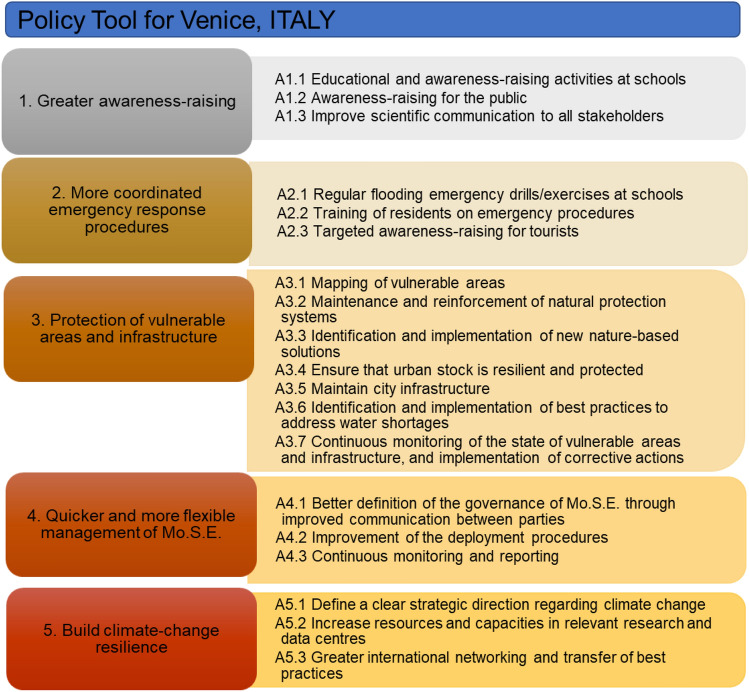
Policy Tool for the Venice Lagoon

The Policy Tool for Venice incorporates the main gaps/needs as well as solutions identified by the stakeholders and builds an Action Plan for adapting and mitigating the impacts of SLR. The Policy Tool includes actions across five main categories: greater awareness-raising, more coordinated emergency response procedures, protection of vulnerable areas and infrastructure, quicker and more flexible management of MoSE and building climate change resilience (Fig. 4).

### Basento, Italy: gaps, needs, suggestions and the policy tool

Stakeholder engagement in Basento involved 7 interviewees and 16 participants at the workshop that took place on 24 March 2022. The SLR vulnerability assessment for Basento resulted in a Moderate-High SLR Vulnerability.

Twelve gaps/needs regarding SLR were identified by the Basento stakeholders, who, during the workshop, decided to work towards the identification of solutions for the two top-ranking needs/gaps. The top-ranking need in Basento was the improvement of urban planning so that it would take SLR into account, an area where most the stakeholders identified a significant gap (Table S6 in Supplementary Material). To bridge this gap the stakeholders proposed the development of integrated strategies for coastal defence that would be linked to an integrated territorial planning strategy. They considered this solution to be the most effective and implementable for improving the area’s urban planning. This is likely in response to the experience of stakeholders with unsuccessful coastal zone management and coastal protection interventions in the area, which had, according to the stakeholders, resulted in “*millions of euros thrown at sea*”. The transformation of the local agricultural crop policy to promote the use of good agricultural practices and the use of crops and varieties that are resilient to climate change was considered by the stakeholders as the second most implementable and effective measure to improve urban planning, whereas the implementation of innovative techniques to protect the coastal zone was also identified as an important solution.

While all the stakeholders noted that they were concerned about SLR and its impacts on the area, they also stated that, even though local people are aware of the ongoing erosion and its effects on the area, awareness about SLR and the link between SLR and erosion is very low. Therefore, the second most important need according to the stakeholders was raising awareness in the local population to ensure that everyone is informed of the causes and impacts of SLR, as well as the means to address it. To address the lack of a common understanding of the issue of SLR, its impacts on the area and the preferred means to address it, the stakeholders proposed the implementation of targeted capacity building, such as for example to civil protection authorities, the provision of information on new, environmentally sound techniques for coastal protection, such as the implementation of nature-based solutions, and knowledge transfer from areas in similar situations that have successfully adapted and mitigated the impacts of SLR.

**Fig. 5:**
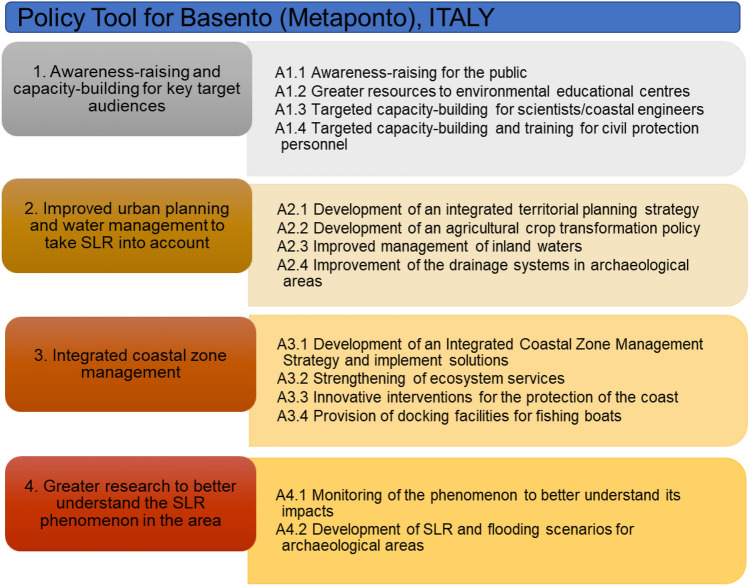
Policy Tool for Metaponto

The importance of raising awareness and building capacity to key stakeholders in the area is also reflected in the developed Policy Tool for Basento (Fig. 5). The Policy Tool is further enriched to include capacity building for scientists/engineers, awareness-raising for the public and the provision of greater resources to environmental education centres to ensure that knowledge and capacity are built in the area’s youth. The need for improved urban planning is also reflected in the Policy Tool where it is enriched with actions related to improving the management of inland waters and improving the drainage systems at nearby heritage sites. Actions are also included to improve the integrated management of the coastal zone that will strengthen ecosystem services, protect the coasts through innovative solutions, such as artificial reefs using environmentally friendly substrates and visually appealing wave attenuators, and ensure that the interests and protection of all stakeholders, are considered. Actions on further research to better monitor the phenomenon and develop scenarios for vulnerable areas including important heritage sites are also included.

### Chalastra, Greece: gaps, needs, suggestions and the policy tool

The stakeholder workshop for Chalastra plain was implemented on 15 April 2022. It was attended by 21 stakeholders. Four stakeholders from the area had been interviewed online. The SLR vulnerability assessment resulted in an Extremely High SLR Vulnerability score for Chalastra, although this was mostly related to the lack of available or accessible data. All the stakeholders in Chalastra expressed a personal feeling of concern with regards to SLR and climate change, although they also noted that while the general problem of climate change is beginning to be considered by decision-makers, “*SLR is not on the agenda yet*” and that decision-makers are concerned by other “*more pressing*” environmental issues such as intensive agriculture, loss of biodiversity and loss of pollinators.

A recurrent theme that emerged from both the interviews and the workshop for Chalastra was a general lack of knowledge of data availability in the area. For example, some stakeholders noted that they had a vague idea that some research was done on the topic but that there was no coordination or dissemination of the relevant data. This is likely the reason why the most important of the 13 needs identified for the area according to the stakeholders is the creation of an observatory for the entire Thermaikos Gulf as a means of facilitating the coordination among relevant bodies and collecting and sharing data regarding climate change and SLR. The measures identified to address this need were the development of a system of monitoring stations around the Thermaikos Gulf, the creation of a GIS portal to share the available data and the development of models and prediction scenarios that would be available to decision-makers (Table S7 in Supplementary Material).

The current impacts of SLR in the Axios Delta/Chalastra region were identified as increased erosion and flooding, although the latter was mostly linked to extreme natural phenomena, such as increased rainfall. Salinisation of aquifers was reported as a suspected phenomenon (viewed in terms of impacts on vegetation) and as a future impact, with potential detrimental effects to biodiversity and agriculture. Groundwater exploitation in the Chalastra plain for agricultural purposes was identified as an exacerbating factor to the impacts of SLR. Therefore, according to the stakeholders, improvements in water management and upgrading of the rainwater drainage system is the second most important need for the area. This need could be met by mapping out the existing rainwater drainage system network, upgrading and supplementing the network using climate change data, and undertaking an investigation of the available water resources and developing suggestions for their sustainable management.

The third most important need identified by the stakeholders concerned the current embankment at Kalochori, located near Chalastra. According to the stakeholders, the embankment, which was developed to prevent marine flooding of the area, needs to be better monitored with regard to its stability and effectiveness. The measures identified were the addition of monitoring sensors along the embankment, the development of prediction scenarios and, if necessary, the redesign of the embankment so it can remain effective, and the creation of groups of volunteers to survey the embankment.

**Fig. 6:**
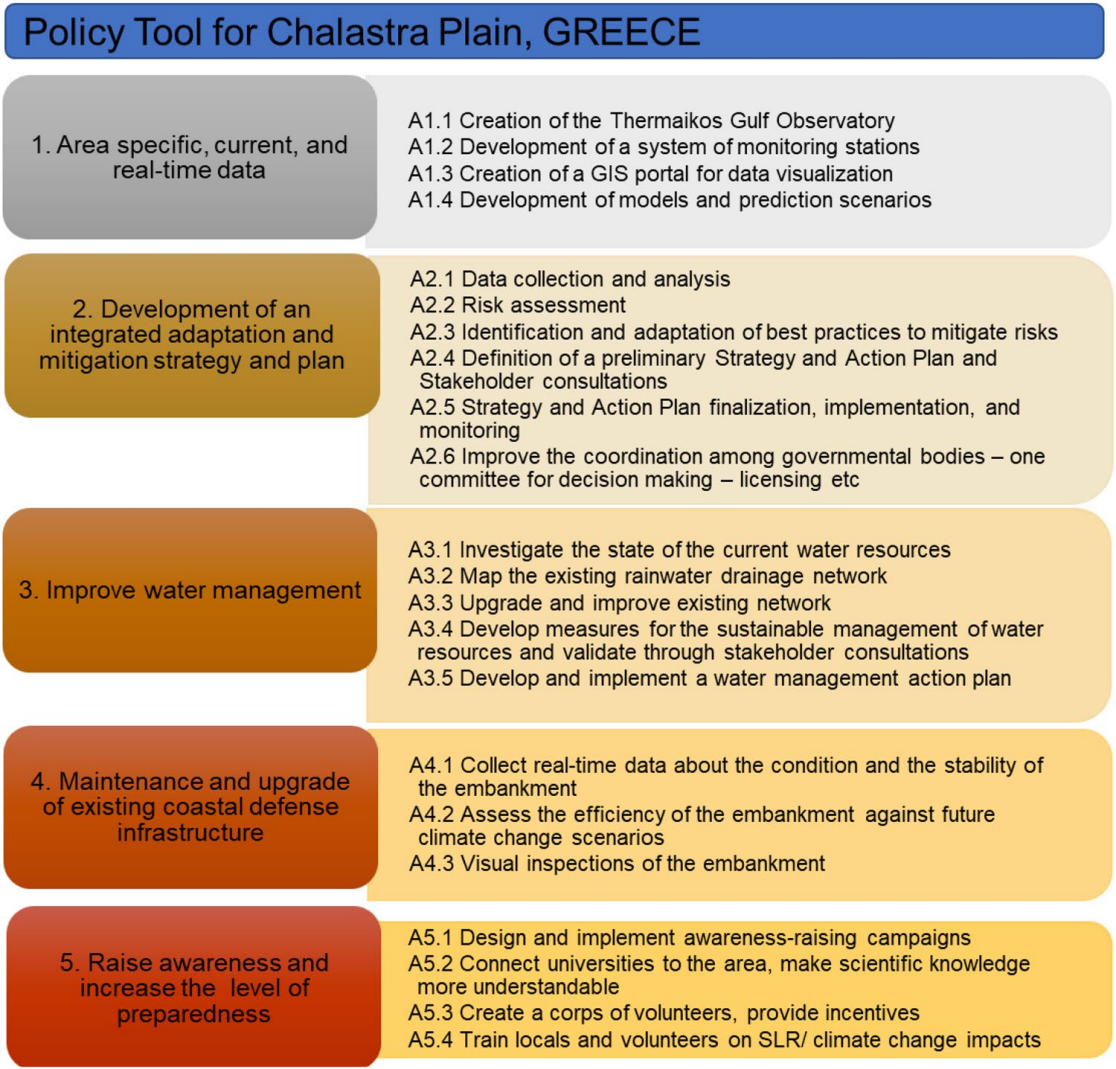
Policy Tool for Chalastra Plain

Finally, the creation of groups of volunteers to raise awareness and respond to emergencies was the fourth need for which stakeholders identified solutions. This need included the launching of a call for volunteers, mainly at universities, connecting universities to the area through the implementation of dissertation/thesis studies, and training volunteers so they have the necessary skills and capacities to respond to climate change and SLR emergencies in the area. The Policy Tool for Chalastra (Fig. 6) reflects the needs and solutions identified by the stakeholders. The creation of a new body, the Thermaikos Gulf Observatory, to oversee the implementation of research work in the area, the collection and sharing of data, and the development of models and scenarios was considered by the stakeholders to be very important, not only for providing much needed data but also for fostering collaboration and coordination between the relevant bodies, including public bodies and scientists. This is closely linked to the second cluster of actions in the Policy Tool on the development and implementation of an integrated adaptation and mitigation strategy based on risk assessments and best practices. The Policy Tool for Chalastra also includes actions that aim to improve overall water management in the area and maintain and upgrade existing coastal defence infrastructures. Finally, actions are also included to raise awareness and increase the level of preparedness in the area, through the incorporation of the solutions identified by the stakeholders and presented above.

### Ebro delta, Spain: gaps, needs, suggestions and the policy tool

Six stakeholders from the Ebro Delta were engaged through interviews. The workshop for the Ebro Delta took place on 5 May 2022 at Deltebre, Spain. It was attended by 19 stakeholders from the area, including representatives of the Consensus Table for the Delta. The latter is a group of seven municipalities, the Delta irrigation communities (i.e. groups of local farmers responsible for irrigation within the Delta as well as for drainage during flooding) and 30 private companies located in the Ebro Delta. The aim of the Consensus Table is to present the local stakeholders’ views and proposals about the management and protection of the Ebro Delta to the Spanish Ministry of Ecological Transition. To this end, the Consensus Table developed the ‘Plan Delta’, which includes several measures for the protection of the area from SLR, erosion and storms. The Consensus Table’s main recommendation is to regulate the use of dams to allow the flow of sediment to the Ebro River delta. The Consensus Table shared Plan Delta with the Ministry of Ecological Transition, receiving the Ministry’s assurance that it would be taken into consideration. However, the Ministry published its own plan, the Plan for the Protection of the Ebro Delta (CEDEX 2021), which does not include the suggestions made by the Consensus Table. The Ministry’s Plan is contentious because one of its recommendations is to convert private land—mainly rice fields—to public land. This has caused significant opposition in the area. As one interviewee stated the feeling in the community is that the Ministry’s plan is “*not a proposal to protect but a proposal for abandonment*”.

It is therefore not surprising that the most important need identified by the Ebro Delta stakeholders was the provision of greater political/decision-making power to the municipalities of the Delta (Table S8 in Supplementary Material). The measures to address this need, according to the stakeholders, are the involvement of local stakeholders in the development of an action plan and supporting environmental impact studies for the management of the area, the creation of a cluster that will include all the local authorities in the area and the effective collaboration between the three levels of government (local, regional and national).

The second most important need identified by the stakeholders was the implementation of solutions for the protection of the coastal area. To do so, the stakeholders suggested that work must primarily focus on an area of 14 km of coast that is particularly vulnerable to the impacts of SLR and climate change. Following that, stakeholders proposed the creation of a natural system of beach, dunes and lagoons in front of the current coastal zone, which could act as a protective buffer for the area, thus protecting the area through nature-based solutions. Increasing the recovery of fluvial systems, currently trapped by the dams used for hydroelectric power generation, was considered as another solution to help minimise erosion and thus protect the coastal zone.

The provision of greater funding for the implementation of innovative solutions, including nature-based solutions, to solve the issue of subsidence and erosion was the third most important need for the Ebro Delta according to the stakeholders. The suitable sources of funding were the hydroelectric power companies through their CSR funds, the European Union, Regional Authorities and the Spanish government.

**Fig. 7:**
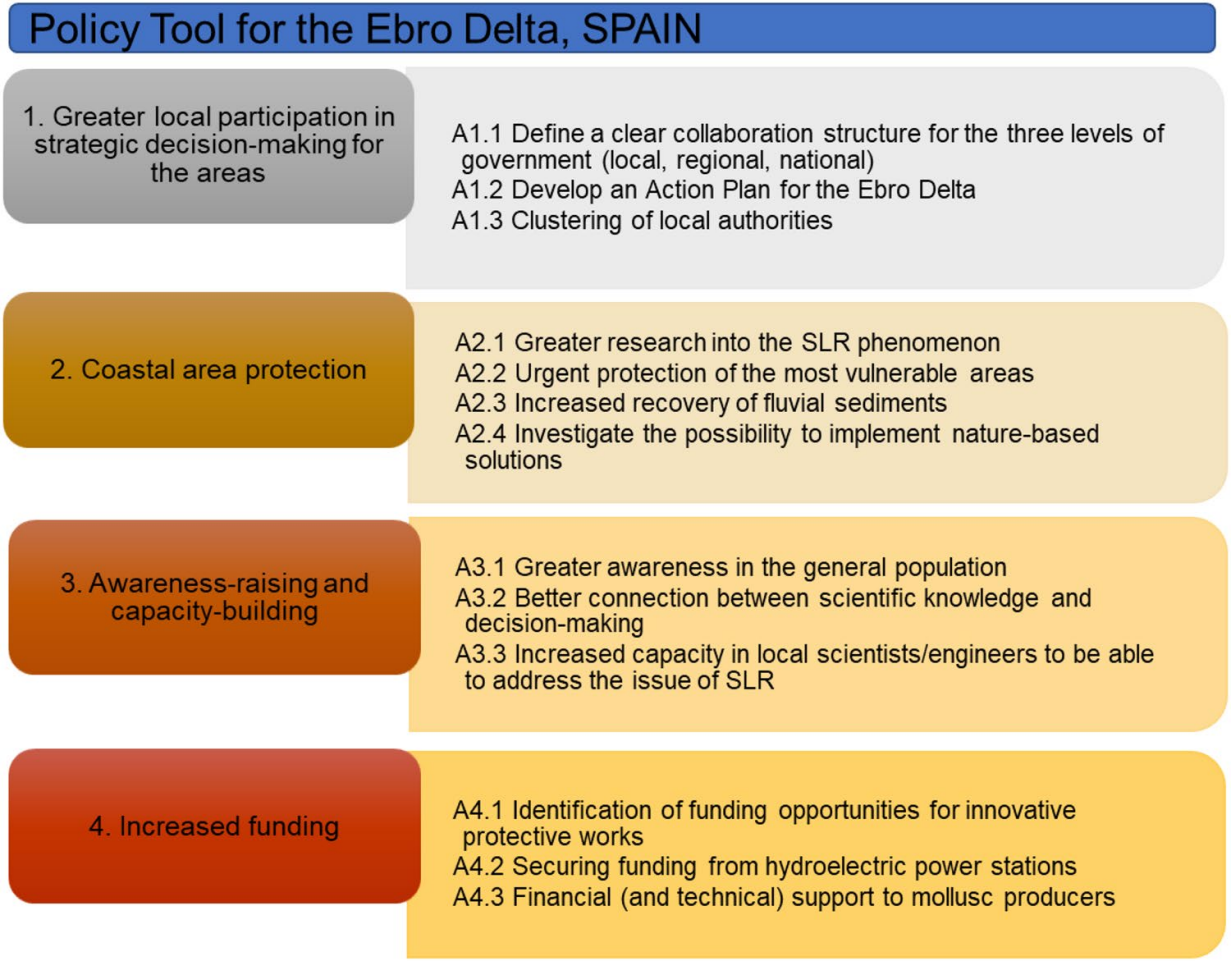
Policy Tool for the Ebro Delta

The Ebro Delta Policy Tool (Fig. 7) includes actions clustered in four groups and aims to address the 10 needs identified by the stakeholders. Further to the solutions discussed above, awareness-raising and capacity building are included in the Policy Tool, addressed through awareness-raising campaigns, ensuring that there is better connection between scientific knowledge and decision-making, and increasing capacity in local scientists/engineers.

## Discussion

The stakeholder engagement presented herein resulted in the development of solution-oriented Policy Tools for each of the four case study areas. The Policy Tools include actions for building SLR resilience closely linked to the specific natural and socio-economic situation of each area. Overall, stakeholders expressed awareness and concern about the current and, even more so, the future impacts of climate change and SLR on their areas, their businesses and their properties. Nonetheless, more research on the topic of SLR and its impacts on the area in question, intensified and more effective awareness-raising campaigns, and improving urban or coastal planning were identified across the four locations as important needs, in line with the previous research on the topic (Stephens et al. 2020; Yusuf et al. 2018). The most important findings of relevance to the definition of SLR adaptation policies in coastal areas are presented below.

### The importance of local-scale scientific data and visualisation tools

An important issue emerging from the study is the necessity for more local-scale scientific information and data on the causes and impacts of SLR, coupled with the “translation” of these scientific data into information that is comprehensive and comprehensible by citizens and stakeholders. Local stakeholders believe that the information provided on SLR vulnerability and risks is too general and refers mainly to global areas, neglecting scenarios and impacts on their local coastal zones. They also feel that the way they receive the information is too scientific and difficult to understand due to a communication gap between scientists and citizens. These two issues distant local stakeholders from SLR knowledge and action.

It is the belief of stakeholders that the disconnect from scientific data on SLR also extends to decision-makers, causing significant gaps in awareness and even major misconceptions. This output is in line with the work of Rizzo et al. (2022) who found that in the Mediterranean there is a need for creating a bridge to allow policy-makers to understand and adopt the results of scientific data and risk assessments regarding SLR. Data are therefore important and useful for a wide range of local stakeholders and high-quality information is important for building credibility and for encouraging communities to act against SLR. However, this information must refer to the specific area (site specific) and be provided in a way that is understandable by stakeholders. A good way of doing this, according our study, is the use of visualisation tools capable of easily showing SLR projections and flooding scenarios up to 2100 AD along the coasts. In fact, the multi-temporal maps of site-specific flooding scenarios for 2030, 2050 and 2100 epochs developed by the SAVEMEDCOASTS-2 project and shared with the stakeholders during the workshops proved to be very useful visual means of transferring scientific knowledge.

### Reluctance to “retreat”

The Policy Tools we co-developed with the stakeholders include actions that fall within the “protect” and “accommodate” adaptation strategies, as discussed by Tol et al. (2008). While in general stakeholders recognised the threats posed by SLR to their vulnerable areas, they were unwilling to suggest or accept solutions within the “retreat” adaptation spectrum. When stakeholders were asked to explain this behaviour, some of them recognised that there is a discrepancy between the “*perception of reality versus the emotional connection to your land and property*”, and noted that there might also be an “*irrational hope and doubting of this problem in fear of loss of tourists, loss of investors and loss of residents*”. Stakeholders from Venice, for example, noted that people tend to normalise the floods caused by extreme high water levels and the phenomenon has been capitalised as a touristic attraction. “*People come to Venice to experience walking barefoot in St Mark’s square*” as one interviewee stated, “*and while stronger events can cause damage to touristic infrastructure, this is something that we are accustomed to and can handle*”. According to the stakeholders, the reluctance to “retreat” could also be attributed to the fact that people tend to accept the risk that comes with living in their area, and that retreat from the area was an unnecessary step and one that would not even become part of the discussion had public bodies seriously considered and implemented other options. For example, in the Ebro Delta, local communities are strongly opposing the government’s proposal for managed retreat, specifically to sacrifice coastal agricultural land to create a buffer zone, considering this to be an act of abandonment on behalf of the government.

### The role of inland and coastal infrastructure

A relevant point emerging from this study is that climate change and SLR should be considered for all infrastructures that might affect the evolution of the coast, including those built inland. The creation of upstream dams and reservoirs in Basento and the Ebro Delta are among the main reasons for significant coastal erosion in these two study areas. Their effects are compounded by SLR and increased frequency and intensity of storm surges, as these were not taken into consideration during their design and construction. Consequently, the proposed solutions in these areas include the improved management of inland waters and fluvial sediments like the reintroduction of fluvial sediments into the delta plain, as suggested by Ibáñez et al. (2014).

The engaged stakeholders were also concerned about maintaining the functionality of existing coastal infrastructures with respect to updated and more accurate information about SLR projections. This was the case of the embankment in Chalastra; stakeholders were concerned about its stability and proposed measures to monitor it. Similarly, in Venice, stakeholders expressed concern about whether updated information and data had been used for the completion of the MoSE. This mobile barrier (https://www.mosevenezia.eu/mose/) that protects the city Venice and its lagoon against extreme high water-level events was designed about 40 years before it became operational, raising doubts about its functionality in view of the latest scientific findings and the recent SLR and climate projections for the next decades and the intensification of meteorological extreme events (Lionello et al. 2021).

### Knowledge gap regarding nature-based solutions

Coastal protection in the case study areas to date mostly involved hard infrastructure, such as breakwaters and barriers, which often resulted in collateral negative impacts such as downdrift erosion. The implementation of more innovative and nature-based solutions for the protection of the coastal zone was a common need identified by the stakeholders in the investigated areas. Yet, this study identified an important lack of awareness of alternatives to hard infrastructure among stakeholders from non-scientific backgrounds. As a result, they initially proposed solutions such as dykes and breakwaters, even though they were contradictory to their views on the area’s aesthetic and future development. When stakeholders from scientific backgrounds suggested softer, nature-based solutions the non-scientific stakeholders agreed that those would be preferable. This indicates that there is a very important information and knowledge gap that needs to be filled so that local stakeholders can understand scientific solutions for coastal protection and thus effectively contribute to the decision-making process. This knowledge gap also affects policy-makers. As one of the stakeholders in Chalastra noted “*Public bodies are still thinking about hard infrastructure as means to address climate change, coastal erosion and so on, whereas they should be thinking about natural based solutions and natural climate buffers*”. It is therefore important to raise awareness about nature-based solutions in decision-makers to ensure support and promotion of such practices.

### Lack of political will to act

According to the engaged stakeholders, the political will to act against climate change and SLR is missing, as economic and political interests very often supersede environmental emergencies. One stakeholder from Basento said, “*The Region thinks about the next election instead of the population, whereas the local politicians think of the next tourist season”* (referring to the expected profit from tourism). In fact, the lack of political and public support and conflicting ecological and social priorities have been identified as major barriers associated with planning and decision-making relating to SLR (Stephens et al. 2020). Because adaptation requires longer-term strategies while politicians work in short time horizons (e.g. the duration of their political mandate), political decision-making becomes a slow process, made even slower by the required collaboration and coordination of multiple administrations (Romagosa and Pons 2017).

The stakeholder engagement method we implemented can help address the lack of political will to act. Through their participation in the decision-making workshops, stakeholders had the opportunity to hear different views and perspectives, to come across new information, and discuss with experts about new techniques/solutions available. This process contributed to social learning and knowledge co-production through meaningful discussions that facilitated the appreciation of other’s views and mutual understanding, resulting in consensus building. The co-creation process ensured that the resulting Policy Tools were accepted and supported and that stakeholders, including participating politicians, became “owners” of the identified solutions, thus increasing their propensity to act for their implementation.

## Conclusions

In this study, we have identified the perceptions on SLR and site-specific mitigation and adaptation practices of key stakeholders in four vulnerable areas of the Mediterranean coasts, in Italy, Greece and Spain. Our participatory method, which included the implementation of interviews and facilitated decision-making workshops, resulted in the development of solution-oriented Policy Tools with actions that are relevant, effective, implementable and stem from stakeholder consensus in each of the investigated areas.

The “translation” of environmental and socio-economic data into SLR vulnerability scores was instrumental in facilitating stakeholders, particularly those from non-scientific backgrounds, to identify where interventions were necessary for minimising their area’s risk, and thus make informed decisions on relevant solutions. The implementation of the workshops in the local language (through the use of interpreters, where necessary) allowed stakeholders to fully participate and express their opinions confidently, in their own language. Our approach can be used for different complex environmental and socio-economic issues where evidence-based decision-making is paramount but where not all the stakeholders might have the necessary background and expertise to understand and interpret the data.

We evidenced a gap in knowledge of not only the issue of climate change and SLR but also of available, best-practice solutions to adapt to it, especially among non-scientific stakeholders and decision-makers. This poses a significant barrier in the definition of site-specific SLR adaptation practices and policies. The participatory stakeholder process employed in this study can be an important vehicle for bridging this gap by fostering knowledge exchange and social learning. This process can also lay the foundations for more extensive participation in public processes through which the resulting Policy Tools can materialise into collectively accepted, concrete actions to help vulnerable areas to adapt to the expected SLR and consequent coastal hazards by the end of this century.

## Supplementary Information

Below is the link to the electronic supplementary material.

Supplementary file1 (DOCX 37 KB)
