# Supplementary material for: Adapting to sea level rise: participatory, solution-oriented policy tools in vulnerable Mediterranean areas
Source: Environ Syst Decis. 2023 Jun 1:1–19. Online ahead of print. doi: 10.1007/s10669-023-09910-5 (PMC10234231; doi:10.1007/s10669-023-09910-5)
Supplement: Supplementary file 1 — Supplementary file1 (DOCX 37 KB) [file 10669_2023_9910_MOESM1_ESM.docx]

**Supplementary Material**

Manuscript "Adapting to sea level rise: participatory, solution-oriented Policy Tools in vulnerable Mediterranean areas”

Submission ID 2cb11963-5e32-4bf6-a258-b8202c3294bb

**Table S1.** The ten stakeholder categories and sub-categories identified in this study. In the Table, i) sectors affected by SLR in the Mediterranean coastal zones, ii) sectors that can (directly or indirectly) impact policymaking and decision-making with regards to SLR in their area, and iii) sectors that have a role to play in raising awareness about SLR, are reported.

| **No.** | **Main stakeholder category** | **Sub-categories** |
| --- | --- | --- |
| 1. | Government and/or policy makers | Local Authority Representatives |
|  |  | National Government - representatives from departments/sectors dealing with climate change, tourism, industry and commerce, city/town planning |
|  |  | Parliamentarians |
| 2. | Coastal and/or marine industry | Port Authorities and Management of commercial ports, fishing ports/shelters, marinas etc. |
|  |  | The tourism sector e.g. tourist agents, owners of hotels, bars, restaurants |
| 3. | Environmental organizations | NGOs, civil society organisations and groups |
| 4. | Education/ Academia / Research | Teachers in Primary & Secondary Schools |
|  |  | Scientists, Researchers (Universities and research centers) |
|  |  | Tertiary Education e.g. professors teaching geography, engineering (civil, coastal, environmental), construction etc. |
|  |  | Relevant scientific networks focusing on the topic of SLR |
| 5. | Commercial/ industrial representatives | Chambers of Commerce |
|  |  | Tourism Associations/Organisations, Regional Development Authorities |
|  |  | Other Business Associations relevant to the site |
| 6. | Insurance sector | Insurance Brokers |
|  |  | Insurance Associations |
| 7. | The media | Newspaper |
|  |  | Radio |
|  |  | Television |
|  |  | Online/web |
| 8. | Various Professional Sectors | Engineers working in the marine/coastal environment |
|  |  | Architects, Civil Engineers |
|  |  | Property Managers active in vulnerable areas |
|  |  | Real Estate Agents working in vulnerable areas |
|  |  | Urban/Environmental Planners |
|  |  | Developers and Construction Professionals |
| 9. | Civil Protection | Coastguard |
|  |  | Firefighters |
|  |  | Other emergency services and Civil Protection Organizations |
| 10. | Utility Providers | Electricity Authority Representatives |
|  |  | Water Boards |
|  |  | Power Station Representatives esp. for those stations located by the coast |

**Table S2** Pillars, indicator categories and indicators included in the DeCyDe-4-SLR factsheet.

| Pillar | Indicator Category | Indicators |
| --- | --- | --- |
| 1. Natural Environment | 1.1 Coastal Area Characteristics | 1.1.1 Average coastal elevation |
|  |  | 1.1.2 Average coastal slope |
|  |  | 1.1.3 Average land subsidence |
|  |  | 1.1.4 Average rate of erosion |
|  | 1.2 Extreme Events | 1.2.1 Frequency of extreme events |
|  | 1.3 Water Resources | 1.3.1 Presence of aquifers |
|  |  | 1.3.2 Presence of river mouths |
| 1. Socio-economic Parameters | 2.1 Resident Population | 2.1.1 Extent of the area |
|  |  | 2.1.2 Resident population |
|  |  | 2.1.3 Annual tourist density |
|  |  | 2.1.4 Tourism’s contribution to the area’s GDP |
|  | - 1. Land Use | 2.2.1 Percentage of the area that is built-up |
|  |  | 2.2.2 Percentage of the area that is used for agriculture/ farming |
|  |  | 2.2.3 Percentage of the area classified as of high ecological value |
|  | 2.3 Infrastructure Vulnerability | 2.3.1 Presence of utility infrastructure in the area |
|  |  | 2.3.2 Presence of hazardous facilities in the area |
|  |  | 2.3.3 Presence of important emergency infrastructure in the area |
|  |  | 2.3.4 Presence of vulnerable infrastructure |
|  |  | 2.3.5 Whether the economic impact of damages to the are related to climate change has been calculated and if so, what percentage of the local GDP it is estimated to be |
|  | 2.4 Cultural Heritage | 2.4.1 Presence of UNESCO world heritage sites in the area |
|  |  | 2.4.2 The distance of these sites from the coast |
| 1. Level of Preparedness | 3.1 Preparedness | 3.1.1 Whether adaptive policies/ strategies for climate change and SLR are in place |
|  |  | 3.1.2 Whether relevant emergency plans are in place |
|  |  | 3.1.3 Whether a climate change or SLR specific risk assessment has been undertaken |
|  |  | 3.1.4 Whether community awareness has taken place |

**Table S3** References used for the scoring ranges in certain DeCyDe-4-SLR indicators

| Indicator | Reference |
| --- | --- |
| 1.1.1 Coastal elevation | Torresan *et al.*, 2012 |
| 1.1.2 Coastal slope | Thieler and Hammar-Klose, 2000 |
| 1.1.3 Land subsidence | Di Paola *et al.*, 2018 |
| 1.1.4 Coastal erosion | Thieler and Hammar-Klose, 2000 |
| 2.1.2 Resident population density | Briggs *et al.*, 2007 |
| 2.1.3 Tourism density rate | Batista e Silva *et al.,* 2018 |
| 2.2 Land use | Benassai *et al.*, 2015 |

**Table S4** Sectors represented by the involved stakeholders (interviews and workshops). A total of 98 relevant stakeholders were involved in the participatory process carried out in this study for developing the solution-oriented policy tools for SLR in the four investigated areas of Venice and Basento in Italy, Chalastra in Greece and the Ebro Delta in Spain.

| Stakeholder Category | Venice, Italy | Basento, Italy | Chalastra, Greece | Ebro Delta, Spain |
| --- | --- | --- | --- | --- |
| Government/ Policy Makers | - Metropolitan City of Venice - Venice City Council - Interregional Supervisor for Public Works for Veneto-Trentino Alto Adige-Friuli Venezia Giulia | - Basilicata Region - Rotondella Local Council | - Thessaloniki Municipality - National Parliament | - Sant Carles de la Ràpita Local Council - Deltebre Local Council - Sant Jaume d’Enveja Local Council - Camarles Local Council - Riumar Council |
| Coastal/ Marine Industry | - Venice Port Authority (Harbour system authority of the of the northern Adriatic Sea) | - Tourist Agents of Metaponto (Leucippo Association) - Network of Tourism Businesses of the Italian Ionic Coast (Rete Turismo Italia Ionica) | - Thermaikos Gulf Protected Areas Management Authority - Navy Captain |  |
| Environmental Organizations | - Marine Archaeology Research Venice (MARVE - NGO) | - Flag Coast to Coast - WWF Italia ‘Bosco Pantano di Policoro’ | - iSea NGO - Biotope/ Wetland Centre | - Grup verd l’Omeda NGO - Park Rangers (Agents Rurals del Montsià) |
| Education/ Academia/ Research | - Tidal Forecasts and Early Warning Centre, City of Venice - ISMAR-CNR (Institute of Marine Sciences) - Cà Foscari University | - CEA Bernalda and Metaponto (Centre for Environmental Education) | - Aristotle University of Thessaloniki | - Eurecat Technology Centre - Centre for Climate Resilience of Catalunya |
| Commercial / Industrial Representatives | - Confartigianato, Venice (Representative of small businesses) - Tourist Guide Association - Guide Sostenibili (Sustainable Tour Guides) |  |  | - Federation of mollusc producers of the Ebro Delta - Community of Irrigators of the Right Ebro Canal |
| The Media | - Il Gazzettino |  |  |  |
| Various Professional Sectors | - Ecoingegno | - Order of Geologists of Basilicata - Basilicata Regional Federation of the Construction Sector | - Civil Engineers - Hydrologists |  |
| Civil Protection |  | - Civil Protection Association |  | - Civil Protection of the Generality of Catalunya |
| Utility Providers | - Veritas S.p.A. |  |  |  |
| Other |  | - Archaeological Park of Herakleia - National Archaeological Museums of Metaponto and Policoro - National Rescue Society |  | - Consensus Table of the Ebro Delta - Hunters Society of the Ebro Delta - Rural Women’s Association - Grievance Ombudsman of Deltebre |

Table S5 Needs/gaps and solutions identified and ranked by the stakeholders of Venice Lagoon

| Top Needs and Solutions | |
| --- | --- |
| Needs (ranked) | **Solutions (ranked)** |
| More information, education, and training, especially regarding what to do in case of emergencies | 1. More training, awareness raising and drills (especially at schools) 2. Best practices for mitigating climate change 3. Training of citizens on emergency plans |
| Quicker and more flexible management of MoSE to ensure that the barriers are active for as little time as possible | 1. Better governance definition 2. Improved procedures 3. Improved communication |
| Protection of vulnerable areas from all threats including erosion | 1. Reinforcement of natural defenses 2. Study on best solutions to address water shortage 3. Implementation of Nature-Based Solutions |
| Other Needs (ranked) | |
| Multi-sectoral collaboration to ensure clarity with regards to the strategic direction of the city as it concerns climate change | |
| Increase the resources and capacities of all relevant research and data centres to ensure increased transfer to policy-makers and citizens | |
| Increased financing to ensure that all structures (including businesses, schools, etc.) are safe and functional | |
| Increased financial resources to ensure the maintenance and protection of infrastructure | |
| Greater research and innovation | |
| Strategy for the management of alarm systems and instructions on how to behave in case of emergencies. This includes the development of an emergency plan for tourists | |
| Lack of sufficient and sustainable clean water supply | |
| Greater international networking and transfer of best practices | |
| Additional interventions, other than the MoSE | |
| Improve scientific communication to all stakeholders at all levels, including the media and citizens | |
| Historical studies and transfer of best practices | |
| Precise information leading to better data and fewer uncertainties | |

Table S6 Needs/gaps and solutions identified and ranked by the stakeholders of Basento, Italy

| Top Needs and Solutions |  |
| --- | --- |
| Needs (ranked) | **Solutions (ranked)** |
| Improved urban planning that takes SLR into account | 1. Integrated strategies for coastal defense 2. Agricultural crop transformation policy 3. Innovative coastal protection techniques |
| Greater awareness raising. Establish a ‘zero point’ i.e. a common basic awareness of the phenomenon, its impacts and how to address it | 1. Targeted capacity-building 2. Information on new techniques for coastal protection 3. Knowledge transfer |
| Other Needs (ranked) | |
| Better monitoring of the area for illegal environmental destruction, and sensitisation so people report illegal actions | |
| Better management of inland water | |
| More resources to environmental education centres | |
| Greater funds for suitable interventions | |
| Strengthening of ecosystem services | |
| Innovative structural interventions | |
| Development of SLR and flooding scenarios for archaeological areas | |
| Understanding of the issue and monitoring of the phenomenon | |
| Improvement of draining systems in Metaponto archaeological areas | |
| Docking facilities for fishing boats | |

Table S7 Needs/gaps and solutions identified and ranked by the stakeholders of Chalastra Plain, Greece

| Top Needs and Solutions |  |
| --- | --- |
| Needs (ranked) | **Solutions (ranked)** |
| Better monitoring of SLR in the entire Thermaikos Gulf, available data (e.g. GIS), and better coordination among relevant bodies. | 1. Monitoring stations across the Gulf 2. GIS portal to hold all data 3. Models and scenarios regarding SLR |
| Improvements in water management and upgrading of the rainwater drainage system | 1. Upgrade network 2. Log existing network 3. Water resources management |
| Monitoring of the stability of the existing embankment | 1. Monitoring sensors 2. Prediction scenarios 3. Volunteers for surveillance |
| Creation of volunteer groups to raise awareness and respond to emergency situations related to flooding, SLR, storm surges and extreme weather conditions | 1. Call for volunteers 2. Training of volunteers 3. Connect universities to the area |
| Other Needs (ranked) | |
| Water saving measures for agriculture and urban areas and valorisation of treated wastewater | |
| Constant interaction between the area’s stakeholders | |
| Implementation of the law | |
| Collaboration with relevant ministries and civil defence to ensure that there is a holistic, joint design of potential adaptation and mitigation actions | |
| Networking, extroversion, and exchange of best practices with sites/authorities at European level | |
| Knowledge/awareness-raising of the problem for the public | |
| Educate and raise awareness both in local stakeholders and in public bodies responsible for the management of the area | |
| Restoration of the wetlands | |
| Increase urban green spaces | |

Table S8 Needs/gaps and solutions identified and ranked by the stakeholders of the Ebro Delta, Spain

| **Top Needs and Solutions** | |
| --- | --- |
| **Needs (ranked)** | **Solutions (ranked)** |
| Greater decision-making power to the municipalities of the Delta | 1. Action plan and environmental impact studies 2. Development of the Ebro Authority 3. Collaboration among government bodies |
| Coastal area protection solutions | 1. Protection of vulnerable 14 km of coast 2. Creation of a natural system in from of the current coast 3. Recovery of fluvial sediments |
| More funding towards real and innovative solutions to the issues of subsidence and erosion | 1. Compensation from hydroelectric companies 2. Funds from the European Union, State and the Region 3. Funds for the annual maintenance of the coastline |
| **Other Needs (ranked)** | |
| Allow sediment to flow from the dams to the coast | |
| Financial and technical support to mollusc producers to adapt to SLR | |
| Change the management of the Delta to confront companies that profit from the dams (hydroelectric power stations) | |
| More research on the topic of SLR | |
| Better connection between scientific knowledge and decision-making | |
| Human resources qualified to help with the issue of SLR | |
| Greater awareness-raising for local decision-makers and the public | |
